# Laboratory Biochemical and Hematological Parameters: Early Predictive Biomarkers for Diagnosing Hepatitis C Virus Infection

**DOI:** 10.1002/jcla.25127

**Published:** 2024-11-21

**Authors:** Saeede Bagheri, Ghazaleh Behrouzian Fard, Nasrin Talkhi, Davoud Rashidi Zadeh, Naser Mobarra, Seyedmahdi Mousavinezhad, Fatemeh Mirzaeian Khamse, Mahdi Hosseini Bafghi

**Affiliations:** ^1^ Department of Laboratory Sciences, School of Paramedical and Rehabilitation Sciences Mashhad University of Medical Sciences Mashhad Iran; ^2^ Department of Biostatistics, School of Allied Medical Sciences Shahid Beheshti University of Medical Sciences Tehran Iran; ^3^ Department of Microbiology, Faculty of Basic Sciences, Mashhad Branch Islamic Azad University Mashhad Iran; ^4^ Department of Laboratory Sciences, School of Paramedical Sciences Sabzevar University of Medical Sciences Sabzevar Iran

**Keywords:** biochemical parameters, HCV infection, hematological parameters, inflammation, predictive biomarkers

## Abstract

**Background:**

Hepatitis C virus (HCV) infection is a worldwide concern, causing liver damage and necessitating early detection to prevent its spread. Studies indicate that evaluating changes in biochemical and hematological parameters, which serve as suitable predictors of inflammation, can be a reasonable method for diagnosing hepatitis C infection.

**Methods:**

This study analyzed 100 samples from high‐risk patients positively identified via quantitative real‐time PCR (qPCR). Anti‐HCV titers, biochemical and inflammatory tests, and complete blood cell counts (CBCs) were performed for these individuals. Additionally, 100 HCV‐negative individuals with normal laboratory results were selected as the control group. Receiver operating characteristic (ROC) curves were plotted to determine the cutoff values of the laboratory parameters.

**Results:**

According to the findings, the age, average white blood cell (WBC) count, platelet‐to‐lymphocyte ratio (PLR), erythrocyte sedimentation rate (ESR), C‐reactive protein (CRP), lactate dehydrogenase (LDH), total bilirubin (TBIL), direct bilirubin (DBIL), alkaline phosphatase (ALP), serum glutamic‐pyruvic transaminase (SGPT), and ferritin levels were significantly higher in HCV patients. On the other hand, red blood cell (RBC) counts, neutrophils, lymphocytes, hemoglobin‐to‐platelet ratio (HPR), and iron (Fe) levels were significantly lower in the case group compared to those in the control group (*p* < 0.05). Furthermore, the ROC curve analysis revealed that lymphocyte count, neutrophil count, and PLR were very strong predictors for hepatitis C infection (*p* < 0.0001, AUC = 1).

**Conclusion:**

The study highlights significant biochemical and hematological differences between HCV patients and healthy subjects. These biomarkers are crucial for early diagnosis, potentially preventing liver damage and reducing HCV transmission.

## Graphical abstract

Hepatitis C virus (HCV) infection is a global concern that can lead to liver damages such as liver fibrosis, liver cirrhosis, or hepatocellular carcinoma in infected individuals. Hundred samples were collected from high‐risk patients who tested positive in quantitative real‐time PCR (qPCR) tests; and anti‐HCV titers, biochemical and inflammatory tests, and complete blood cell counts (CBCs) were performed for these individuals. Our results indicated significant differences between hepatitis C patients and the healthy control group.

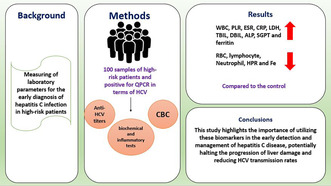

## 1 Introduction

HCV infection is recognized as a major liver disease worldwide, affecting both developed and developing countries [1, 2]. This flavivirus is structurally enveloped and possesses a single‐stranded RNA that encodes a single polyprotein. Currently, there is no effective vaccine against it [3]. The main risk factors for infection include the use of injectable drugs, contact with contaminated equipment, and receiving blood transfusions from unscreened individuals [4]. The pathogenesis of the infection involves the activation of cytotoxic T lymphocytes, which, in their effort to combat the virus, destroy the infected liver cells and cause liver damage [5]. To date, seven genotypes of HCV have been identified, each showing certain geographic variations in prevalence [6, 7]. Approximately 71 million people worldwide are infected with the virus, and acute HCV infections often lead to chronic, long‐term infections [8]. These individuals are susceptible to cirrhosis and liver cancer, with approximately 350,000 deaths annually attributed to complications of HCV [9]. Although most people with acute HCV infection are asymptomatic, up to 30% of them become symptomatic [10]. These symptoms may include weakness, anorexia, right upper quadrant abdominal pain, dark urine, spider angioma, lower extremity edema, thrombocytopenia, and jaundice, along with SGPT and serum glutamic‐oxaloacetic transaminase (SGOT) levels greater than 10 times the normal range, indicating liver tissue damage [11]. SGPT, as a cytosolic enzyme, easily enters the blood circulation following hepatocellular damage and is considered a sensitive marker of liver damage [12], whereas SGOT is not specific to the liver [13]. Nevertheless, a combined approach considering the AST/ALT ratio may be useful in monitoring patients with HCV [14].

However, 30% of patients with acute HCV infection spontaneously clear the infection within 6 months [11, 15]. On the other hand, HCV is the primary cause of liver fibrosis, liver cirrhosis, and hepatocellular carcinoma (HCC) in patients [16]. Additionally, several extrahepatic manifestations are associated with chronic HCV infection, including insulin resistance, type 2 diabetes, cardiovascular disease, and chronic kidney disease (CKD). A meta‐analysis has shown that chronic HCV infection is associated with a 50% increased risk of proteinuria and a 40% increased incidence of CKD [17]. Therefore, early diagnosis of this virus is essential to treat and prevent disease progression, as well as to prevent further transmission, considering the severity of conditions caused by HCV.

PEGylated‐interferon‐alpha (peg‐IFN‐α) and ribavirin have been used as a combination therapy for HCV infection [18]. Today, several direct‐acting antiviral (DAA) drugs have replaced interferons as the standard treatment for HCV infection [19]. According to their mechanisms of action and therapeutic goals, these antivirals are categorized into four groups: NS3/4A protease inhibitors, NS5A replication complex inhibitors, and NS5B nucleoside and non‐nucleoside polymerase inhibitors [20, 21]. Among the NS5B nucleoside polymerase inhibitors, sofosbuvir (SOF) is the primary drug that plays a vital role in combination with other DAAs for the treatment of HCV [22].

The recombinant immunoblot assay (RIBA) and nucleic acid amplification test (NAT) are two tests known for their high specificity in detecting HCV. However, these tests are not cost‐effective due to their high expenses [23]. Additionally, the recommended diagnostic approach for HCV infection involves preliminary screening using a serological assay for HCV antibody, followed by molecular testing for HCV RNA to confirm the presence of the HCV [24]. Typically, guidelines advocate for the use of a reliable serological diagnostic test such as ELISA (EIA) or chemiluminescence‐based immunoassay in laboratory settings. Rapid diagnostic tests for detecting HCV antibodies are also designed to meet minimum performance standards [25]. On one hand, since hepatitis C infection is characterized by chronic inflammation [26] and hemogram parameters have been utilized as markers of inflammation in various conditions [27, 28], the significance of employing these parameters in the diagnosis of HCV can also be emphasized. For example, it has been reported that the PLR, which is derived from hemogram parameters, is associated with inflammatory diseases, including gastrointestinal disorders [29], irritable bowel syndrome [30], thyroid issues [31], thyroiditis [32], cancer [33], diabetes mellitus [34], and COVID‐19 infection [35]. The neutrophil‐to‐lymphocyte ratio (NLR) is also a new inflammatory marker extracted from routine blood tests. Its connection to inflammation has been reported in diabetes mellitus [36], cardiac conditions [37], and thyroiditis [38]. Additionally, markers derived from hemoglobin have been suggested as predictors of inflammation in inflammatory conditions [39], making the study of these markers in hepatitis C infection reasonable.

CBC and biochemical tests are among the most common and readily available blood tests. They are routinely conducted during medical examinations, even for asymptomatic individuals. However, there has been limited evaluation of the information derived from CBC and biochemical tests so far. The aim of this study was to explore laboratory methods and their interrelationships, such as molecular assays, measurement of HCV antibody levels, CBC, and blood indices, as well as liver and inflammatory tests, for the early diagnosis of hepatitis C in high‐risk patients.

## 2 Materials and Methods

This case–control study involved 100 patients (homeless addicts) who were referred to the Médecins Sans Frontières/Doctors Without Borders (MSF) medical and humanitarian aid center in Mashhad, Iran. This center was established to monitor high‐risk patients. Whole blood samples were collected from the patients, and after separating plasma and serum, a qPCR test was conducted to identify patients with hepatitis C and carriers. Subsequently, the positive samples were monitored for HCV infection and virus load using cycle quantification (Cq) values obtained from the qPCR tests [40]. Each patient underwent testing and measurement for HCV IgG and IgM antibodies, CBC, PLR, NLR, and HPR, as well as serum ferritin and Fe. Liver function tests including SGPT, SGOT, ALP, TBIL, DBIL, and inflammatory tests including CRP and ESR were also performed. Additionally, 100 control subjects with normal laboratory parameters were selected for comparison with the case group.

### 2.1 Hematological, Immunological, and Biochemical Tests

The CBC test was carried out utilizing a calibrated cell counter (Sysmex XP 100, Japan). ESR was assessed using an optical reader (Therma 100, Spain). Quantitative CRP levels were determined turbidometrically, while LDH, SGOT, SGPT, and ALP enzymes, TBIL, DBIL, Fe, and ferritin levels were measured using colorimetric methods, employing an auto‐analyzer (OLYMPUS AU640, Japan). Furthermore, anti‐HCV IgG and IgM tests were performed using the ELISA method (Pishtaz Teb Kit, Iran).

### 2.2 Real‐Time PCR Molecular Test

The RNA extraction was carried out using the total RNA column isolation kit from Sacace, Italy. The quality and quantity of the extracted RNA were evaluated using a Nanodrop spectrophotometer (Thermo Scientific, USA) at A260/A280 nm [41]. Complementary DNA (cDNA) was synthesized through reverse transcription employing a Sacace cDNA kit (Italy) and the extracted RNA template [42]. The qPCR for the target genes was carried out using the Roche LightCycler 96 real‐time PCR Instrument (Germany). HCV IC served as an internal control for assessing the sampling and RNA extraction accuracy. The total reaction volume was 25 μL with 12.5 μL for the RNA sample. The reaction mix consisted of 7.5 μL of RT‐PCR‐mix‐1‐ HCV, 5 μL of mix RT‐PCR‐mix‐2‐FRT and dithiothreitol (DTT), 0.5 μL of Taq polymerase, and 0.25 μL of TM‐Revertase Moloney Murine Leukemia Virus Reverse Transcriptase (MMlv). The amplification program included an initial denaturation step at 95°C for 15 min, followed by 40 cycles. Each cycle comprised four steps: denaturation at 95°C for 5 s, annealing and elongation at 60°C for 20 s, and 72°C for 15 s. Calibration curves for each target gene were created using serial dilutions, and the PCR efficiency was calculated to be between 98% and 99%. Negative controls were included in each run [43].

### 2.3 Statistical Analysis

The results were reported as mean and standard deviation (SD). Statistical analysis was carried out using SPSS 22 software. Data were categorized into two groups: the case group and the control group. The normality of data distribution was evaluated using the Kolmogorov–Smirnov and Shapiro–Wilk statistical tests. To compare between groups, the Mann–Whitney test, independent *t*‐test, Wilcoxon test, and chi‐square test were utilized. Pearson and Spearman's tests were used to investigate correlations between parameters. Furthermore, ROC curves were constructed to calculate the parameters' sensitivity and specificity.

## 3 Results

In the present study, 200 participants were included. Following the results of the qPCR test, participants were segregated into two groups: the case group and the control group. Out of the participants, 100 individuals tested positive for the qPCR test, signifying the existence of the HCV in the case group. The gender distribution in the case group was 76% male and 24% female. Diverse parameters such as age, hematological, inflammatory, and biochemical measures were assessed and compared between the case and control groups, and the findings are outlined in Table 1.

**TABLE 1:** The mean and standard deviation of parameters and their comparison between case and control groups.

Parameters	Control mean ± SD	Case mean ± SD	*p*
Age (year)	17.88 ± 15.04	35.24 ± 17.51	0.000
HCV Ab (mIU/mL)	—	7.02 ± 4.61	—
SGOT (mg/dL)	25.73 ± 9.29	46.32 ± 68.81	0.94
SGPT (mg/dL)	19.23 ± 7.54	59.28 ± 91.59	0.000
ALP (mg/dL)	219.77 ± 109.78	313.66 ± 218.22	0.038
LDH (U/L)	238.03 ± 78.50	469.91 ± 239.11	0.000
TBIL (mg/dL)	0.69 ± 0.25	2.50 ± 2.99	0.000
DBIL (mg/dL)	0.17 ± 0.58	2.66 ± 9.40	0.000
CRP (mg/dL)	3.61 ± 4.91	15.86 ± 16.45	0.000
ESR (mm/h)	9.59 ± 3.13	26.65 ± 21.40	0.000
Fe (μg/dL)	104.93 ± 29.83	83.76 ± 44.66	0.000
Ferritin (μg/L)	45.51 ± 22.21	185.97 ± 182.57	0.000
RBC (×10^6^/μL)	4.90 × 10^6^ ± 3.76 × 10^6^	4.56 × 10^6^ ± 9.85 × 10^6^	0.000
HGB (g/dL)	13.47 ± 1.01	13.02 ± 2.59	0.112
Hematocrit (HCT) (%)	39.83 ± 3.05	38.74 ± 7.83	0.194
WBC (×10^3^/μL)	7.00 × 10^3^ ± 1.47 × 10^3^	9.95 × 10^3^ ± 6.05 × 10^3^	0.000
Neutrophil (×10^3^/μL)	5.60 × 10^3^ ± 3.01 × 10^3^	6.20 × 10^3^ ± 5.16 × 10^3^	0.000
Lymphocyte (×10^3^/μL)	3.53 × 10^3^ ± 2.66 × 10^3^	3.44 × 10^3^ ± 2.44 × 10^3^	0.000
Platelet (×10^3^/μL)	236 × 10^3^ ± 431 × 10^3^	280 × 10^3^ ± 110 × 10^3^	0.590
PLR	6.72 ± 1.28	107.47 ± 73.59	0.000
NLR	1.59 ± 0.17	2.43 ± 3.11	0.222
HPR	0.58 ± 0.12	0.56 ± 0.29	0.005

According to the results, the average age and mean values of WBC, PLR, ferritin, ESR, CRP, LDH, SGPT, ALP, TBIL, and DBIL were significantly higher in HCV‐positive cases compared to those in normal cases (*p* < 0.05). Conversely, the average values of RBC, neutrophil, lymphocyte, HPR, and Fe in the case group were notably lower than those in the control group (*p* < 0.05).

### 3.1 Analysis of ROC Curves to Evaluate Hematological and Inflammatory Indices in HCV‐Positive Individuals

**FIGURE 1:**
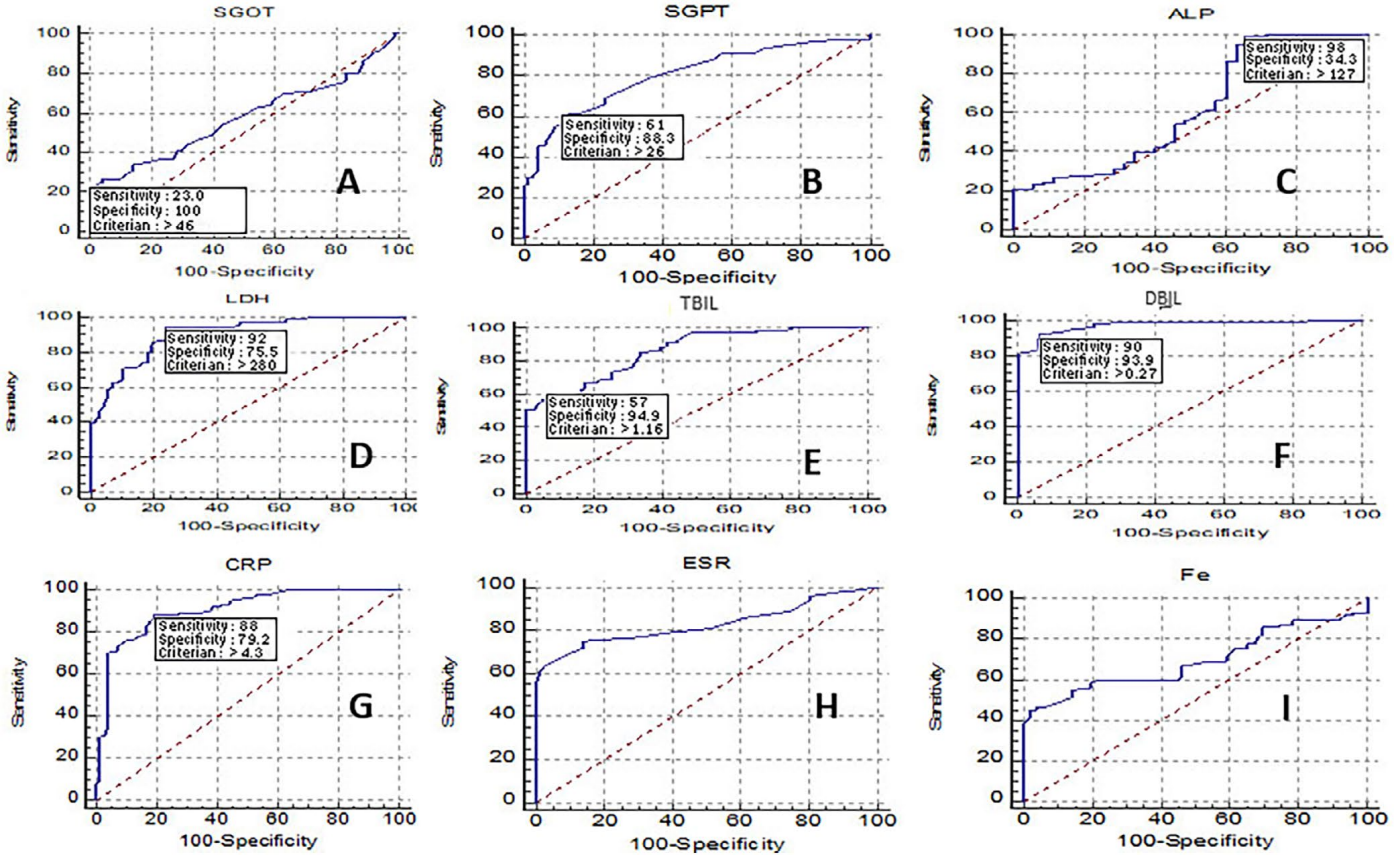
Evaluation of biochemical and inflammatory indices in HCV patients using the ROC curves. The parameters of SGOT (A), SGPT (B), ALP (C), LDH (D), TBIL (E), DBIL (F), CRP (G) and ESR (H), and Fe (I) were statistically significant sensitivity and specificity.

**FIGURE 2:**
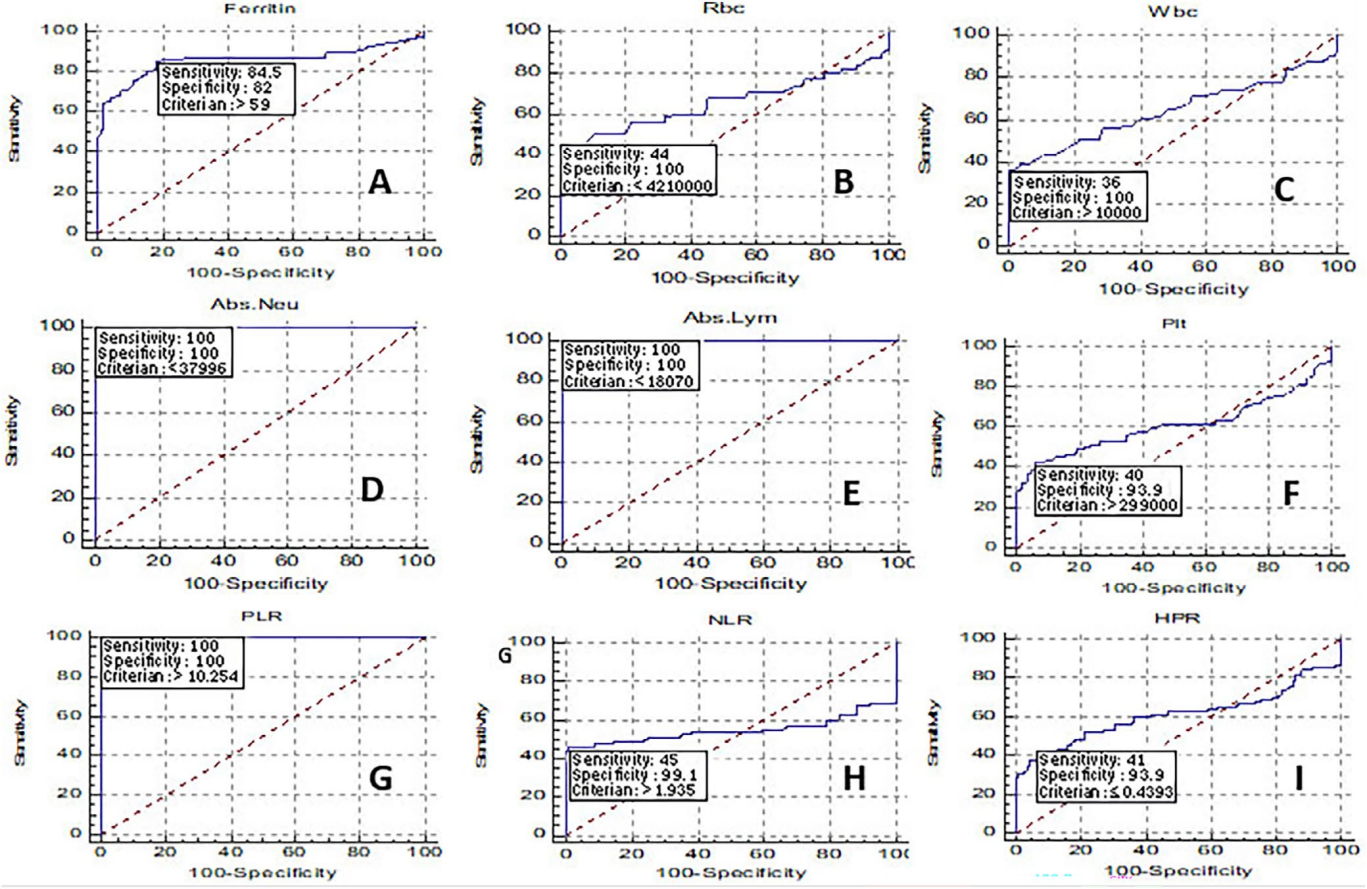
Evaluation of hematological indices in HCV patients using the ROC curves. The parameters of ferritin (A), RBC (B), WBC (C), neutrophil (D), lymphocyte (E), PLT (F), PLR (G), NLR (H), and HPR (I) were statistically significant sensitivity and specificity.

To assess the diagnostic significance of SGOT, SGPT, ALP, LDH, TBIL, DBIL, CRP, and HPR parameters, we performed the ROC curve analysis using data obtained from both patient and control groups. The findings of the ROC curve analysis are detailed in Table 2 and Figures 1 and 2. This study conducted ROC curve analysis to determine optimal cutoff points for the measured parameters in predicting HCV infection. The results indicated that SGPT, LDH, TBIL, DBIL, CRP, ESR, ferritin, neutrophil, lymphocyte, and PLR factors served as reliable predictors for hepatitis C infection (area under the ROC curves (AUCs) = 0.803–1.000).

**TABLE 2:** ROC curve analysis of hematological, biochemical, and inflammatory parameters.

Parameters	AUC	Significance level *p*	95% confidence interval	Sensitivity	Specificity	Cutoff
SGOT (mg/dL)	0.576	0.082	0.498 to 0.651	23.0	100	> 46
SGPT (mg/dL)	0.803	< 0.0001	0.736 to 0.859	61	88.3	> 26
ALP (mg/dL)	0.618	0.049	0.530 to 0.700	98	34.3	> 127
LDH (U/L)	0.902	< 0.0001	0.854 to 0.939	92	75.5	> 280
TBIL (mg/dL)	0.856	< 0.0001	0.799 to 0.902	57	94.9	> 1.16
DBIL (mg/dL)	0.972	< 0.0001	0.938 to 0.990	90	93.9	> 0. 27
CRP (mg/dL)	0.904	< 0.0001	0.855 to 0.941	88	79.2	> 4.3
ESR (mm/h)	0.829	< 0.0001	0.769 to 0.878	75	86	> 12
Fe (μg/dL)	0.694	< 0.0001	0.621 to 0.760			
Ferritin (μg/L)	0.855	< 0.0001	0.796 to 0.902	84.5	82	> 59
RBC (×10^6^/μL)	0.657	0.0001	0.589 to 0.720	44	100	< 4,210,000
HGB (g/dL)	0.556	0.200	0.486 to 0.623	35	99.1	< 11.7
HCT (%)	0.543	0.3341	0.473 to 0.611	38	94.8	0.334
WBC (×10^3^/μL)	0.642	0.0004	0.574 to 0.706	36	100	> 10,000
Neutrophil	1.000	< 0.0001	0.983 to 1.000	100	100	< 37,996
Lymphocyte	1.000	< 0.0001	0.983 to 1.000	100	100	< 18,070
Platelet (×10^3^/μL)	0.607	0.0113	0.538 to 0.672	40	93.9	> 299,000
PLR	1.000	< 0.0001	0.983 to 1.000	100	100	> 10.254
NLR	0.548	0.3020	0.479 to 0.616	45	99.1	> 1.935
HPR	0.611	0.008	0.542 to 0.676	41	93.9	≤ 0.4393

## 4 Discussion

The severity of liver fibrosis resulting from HCV infection correlates with the degree of viral activity, the quantification of which can be achieved through the qPCR molecular testing of plasma samples. A crucial aspect in predicting outcomes, accurate diagnosis, clinical management, and monitoring the impact of HCV infection on patients' health is to conduct additional laboratory investigations. These tests encompass biochemical, inflammatory, and hematological analyses and entail examining their interrelations [44, 45]. The current study's results elucidate the molecular, hematological, inflammatory, and biochemical changes linked to HCV infection in patients and carriers. A key strength of our study lies in the initial confirmation of HCV infection through qPCR molecular testing, followed by subsequent monitoring of the other specified laboratory assessments to assess their correlation in HCV‐positive samples. It is important to highlight that analyzing gene expression profiles and viral loads through molecular testing plays a crucial role in assessing disease stages and treatment progression with antiviral agents [46]. In 2020, JALIL et al. conducted a study to investigate the impacts of various hepatitis viruses (HAV, HBV, and HCV) and the prevalence of infections across different age groups. They collected samples from 75 hepatitis patients (25 women and 50 men) aged 18–65, as well as from a control group of 20 healthy individuals (9 women and 11 men) ranging from 17 to 63 years old. The findings indicated that there was no significant difference in age and gender between the patients and the healthy individuals [47].

In clinical practice, evaluating serum liver enzymes serves as the primary method for assessing liver injury. This process is a vital tool for healthcare practitioners in detecting liver diseases like hepatitis C in their initial stages [48]. In this study, the levels of the SGPT enzyme were notably elevated in hepatitis C patients compared to those in the control group. Aligning with this research, other studies have similarly demonstrated a significant increase in SGPT levels among a majority of hepatitis C patients and 70%–80% of carriers [49, 50]. Ufearo et al. demonstrated that HCV seropositivity is associated with significant increases in SGOT, SGPT, and gamma‐glutamyl transpeptidase (GGT) levels, along with changes in serum protein profiles, suggesting a chronic inflammatory condition, marked by higher globulin levels and reduced albumin concentration [51]. In the study by Cacciola et al., elevated levels of SGOT, SGPT, and GGT were observed in 1189 out of 5806 individuals who attended the surgeries of 14 general practitioners (GPs) working in Messina, Italy (20.5%, mean age 56.4 ± 16.9 years), with no difference in gender distribution (764 out of 3743 males; 425 out of 2063 females, *p* = 0.29) [52]. In our study, the target population consisted of high‐risk drug addicts undergoing HCV screening at the MSF center in Mashhad, Iran.

Furthermore, the current study found significantly elevated ALP levels in patients diagnosed with HCV. Recent research also suggests that increased ALP levels are commonly associated with various medical conditions such as liver metastasis, extrahepatic bile obstruction, primary biliary cirrhosis, intrahepatic cholestasis, infiltrative liver disease, hepatitis, cirrhosis, primary sclerosing cholangitis, hepatic lymphoma, liver abscess, sarcoidosis, and congestive heart failure. Importantly, a deviation in ALP levels exceeding 120 U/L may indicate disease progression [53, 54].

CBC is a commonly used blood test due to its ability to detect changes in peripheral blood cells [55]. Given its sensitivity to subtle blood variations, CBC is routinely carried out during health assessments, even for asymptomatic individuals [56]. In this study, we compared the CBC profiles of HCV‐positive patients with controls, revealing significant differences between the two groups. Specifically, WBC, neutrophil count, and PLR were notably higher in HCV‐positive individuals. The elevation in WBC levels during hepatitis infections, particularly with HCV, is linked to the immune system's response to the virus antigens [57, 58]. On the contrary, RBC, lymphocyte counts, and HPR were notably lower in individuals who tested positive for HCV. However, there were no statistically significant differences observed in HGB, HCT, PLT, and NLR levels between the case and control groups. In line with our research, Dass et al. in 2022 conducted a study in India where they also did not find statistically significant differences in hemoglobin levels between participants who were anti‐HCV‐positive and ‐negative [59]. On the other hand, Tsai et al.'s study in Taiwan analyzed data from 312 blood donors, consisting of 144 anti‐HCV‐positive and 168 anti‐HCV‐negative donors. The results indicated that platelet counts in the group of HCV patients were notably lower than those in the control group [60]. Moreover, Ain et al.'s research in Pakistan in 2022 revealed that thrombocytopenia linked to the administration of common antiviral medications (PEG‐IFN) was observed in patients [61].

It has been suggested that an elevated level of serum ferritin can act as an early indicator of the severity of chronic liver disease, correlating with the degree of hepatic steatosis grading. In this investigation, serum ferritin levels were notably elevated in patients with HCV compared to those in healthy individuals. According to our research, Chang et al.'s findings indicated that individuals infected with HCV exhibited increased levels of ferritin in their bloodstream [62]. In chronic hepatitis C, serum ferritin levels can increase due to HCV‐induced hepcidin depletion [63]. HCV can inhibit hepcidin mRNA in the Huh7.5 cell line, followed by increased hepatic iron, as demonstrated by Liu et al. [64]. The accumulation of iron can contribute to oxidative stress, liver fibrosis, and cirrhosis. Furthermore, research has revealed that hepcidin contributes to reducing HCV replication in the Huh7.5 cell line [65]. Moreover, our results indicated a significantly lower serum Fe level in cases compared to those in controls. This study highlighted notable iron profile findings, showing low serum iron levels and high ferritin levels in HCV patients. These clinical observations suggest the anemia of chronic diseases, in line with the observations of Elmowafy et al. [66].

The ROC curve is a common tool used to evaluate the diagnostic test's ability to discriminate, aiding in the analysis and interpretation of results. This research highlighted that SGPT, LDH, TBIL, DBIL, CRP, ESR, ferritin, neutrophil count, lymphocyte count, and PLR were significant predictors for HCV. The examination and depiction of ROC curves utilizing the available data proved to be a crucial aspect of our current study, potentially distinguishing it from others. Nevertheless, this study's limitations encompass the absence of exploration into antiviral drugs' impact on HCV, as well as the omission of investigating antiviral side effects on hematological, biochemical, and inflammatory parameters and, lastly, the lack of follow‐up on the treatment outcomes for the study cohort. Enhanced screening through increased sample sizes and participant numbers in studies could offer a more comprehensive outlook for monitoring patients and controlling the disease in symptomatic individuals, carriers, and those at high risk.

Overall, it can be said that as research continues to identify viral and host factors associated with the pathogenesis of HCV infection, extrahepatic manifestations, recovery, and treatment response, the evolution of laboratory assays and the application of these parameters for HCV diagnosis also require regular re‐evaluation in the clinical setting. In this regard, defining the purpose of the test and selecting the correct assay are critically important. Consensus conferences that involve experts and specialists from various fields provide an ideal environment for discussing these topics.

## 5 Conclusion

HCV is a leading cause of HCC worldwide. Improved HCV screening methods, which enable the earlier detection of the virus, can not only help reduce its transmission but also significantly decrease the prevalence of chronic HCV cases and, consequently, the incidence of HCC. According to the results of this study, hematological and biochemical markers exhibit significant differences between individuals with hepatitis C and healthy individuals. Key indicators such as increased WBC, PLR, ferritin level, ESR, CRP, LDH, TBIL, DBIL, ALP, and SGPT, as well as a decreased Fe level and RBC, neutrophil, and lymphocyte counts, are associated with HCV. These findings underscore the importance of utilizing these biomarkers in the early detection and management of hepatitis C, potentially halting the progression of liver damage and reducing HCV transmission rates. Furthermore, while the study highlights key diagnostic parameters, certain limitations need to be addressed. There is a lack of data on the effects of antiviral treatments on the identified biomarkers. Additionally, the absence of a long‐term follow‐up on treatment outcomes restricts the ability to evaluate the markers in response to disease progression and treatment. Further research is necessary to validate the utility of these indicators in clinical settings.

## Author Contributions

Saeede Bagheri: investigation, writing – original and editing; Ghazaleh Behrouzian Fard: validation, editing; Nasrin Talkhi: formal analysis, data curation; Davoud Rashidi Zadeh: validation; Naser Mobarra: investigation; Seyedmahdi Mousavinezhad: formal analysis; Fatemeh Mirzaeian Khamse: software; Mahdi Hosseini Bafghi: supervision, project administration, review and editing, validation. All the authors have read and approved the manuscript.

## Ethics Statement

We prioritize human rights, welfare, and privacy. We obtain informed consent from participants, protect their anonymity and confidentiality, and minimize any potential harm or discomfort they may experience because of their involvement. We comply with all relevant laws, regulations, and guidelines governing research ethics. This research has an ethics approval with ID: IR.MUMS.FHMPM.REC.1401.217 issued by the Mashhad University of Medical Sciences, Mashhad, Iran.

## Conflicts of Interest

The authors declare no conflicts of interest.

## Data Availability

The data used to support the findings of this study are available from the corresponding author upon request.
